# Supplementary material for: Saccharomyces boulardii (CNCM I-745) improves intestinal damage in sepsis by remodeling intestinal flora structure
Source: Front Cell Infect Microbiol. 2025 Jul 31;15:1602792. doi: 10.3389/fcimb.2025.1602792 (PMC12350329; doi:10.3389/fcimb.2025.1602792)
Supplement: Supplementary file 1 [file DataSheet1.zip › summary/summary/7_Differential_analysis/1_diff_abundance/5_Family/Group.DW4_vs_DW0/Group.DW4_vs_DW0_diff_barplot.pdf]

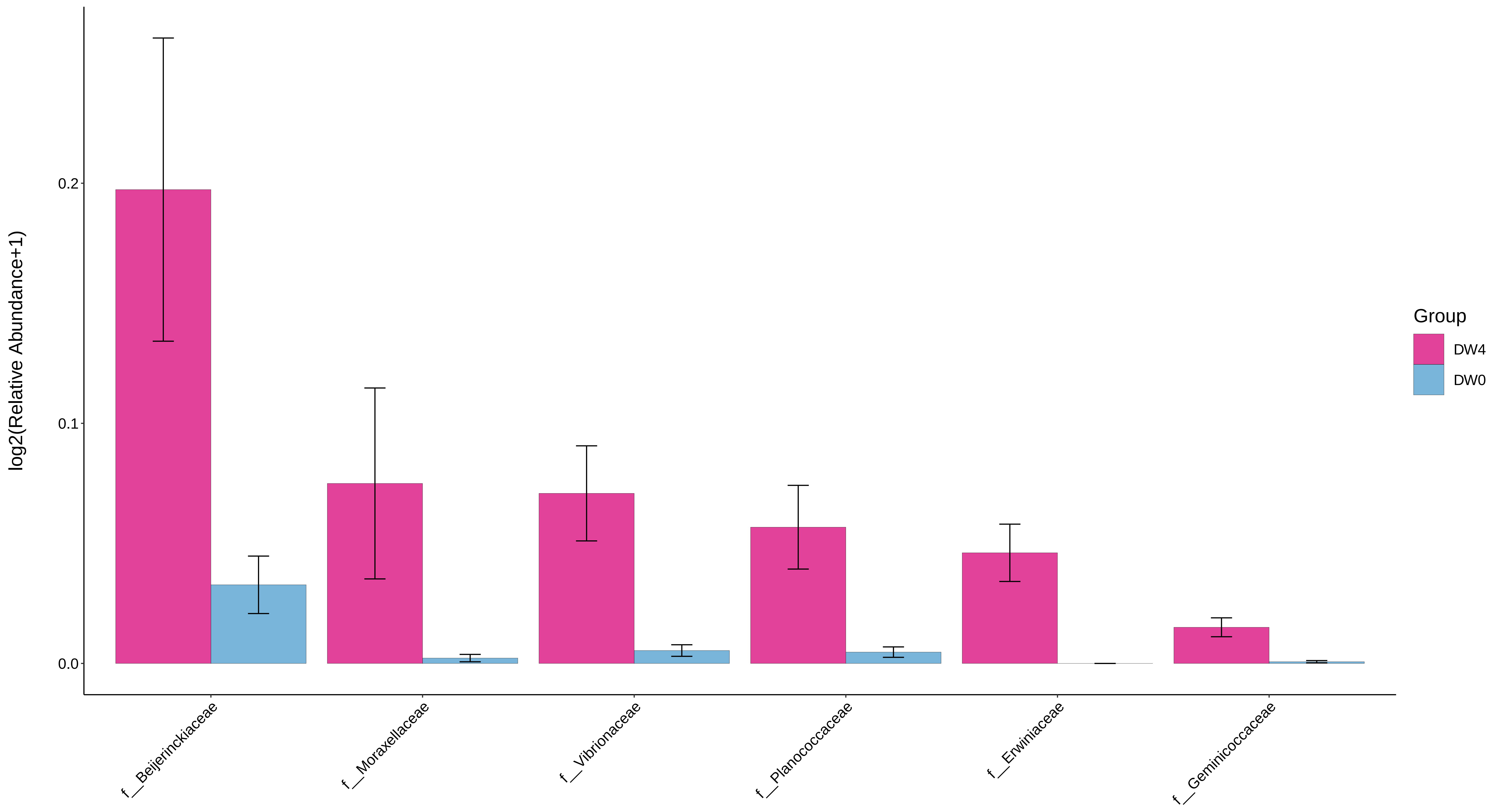

log2(Relative Abundance+1)
0.2
0.1
0.0
f__Beijerinckiaceae
f__Moraxellaceae
f__Vibrionaceae
f__Planococcaceae
f__Erwiniaceae
f__Geminicoccaceae
Group
DW4
DW0
